# Role of Inflammatory Cytokines and Endocrine Dysregulation in Pain-Related Cardiovascular and Metabolic Dysfunction: A Narrative Review

**DOI:** 10.7759/cureus.103034

**Published:** 2026-02-05

**Authors:** Oyebisi M Azeez, Happiness Olaniyi, Japheth O Oyovwi, Mary I Oyovwi, Chinaecherem P Okafor, Adetayo Folasole, Obiageri Ihuarulam Okeoma, Aliyu O Olaniyi

**Affiliations:** 1 Veterinary Physiology and Biochemistry, University of Ilorin, Ilorin, NGA; 2 Nursing, Barton Brook Care Home, Manchester, GBR; 3 General Medicine, Stepping Hill Hospital, Manchester, GBR; 4 General Practice, Bolton NHS Foundation Trust, Manchester, GBR; 5 Biochemistry, The Graduate Center, City University of New York (CUNY), New York, USA; 6 Computing, East Tennessee State University, Johnson City, USA; 7 Medical Laboratory Science, Trinity University Yaba, Lagos, NGA; 8 Geriatrics, Stepping Hill Hospital, Manchester, GBR

**Keywords:** cardiovascular dysfunction, chronic pain, endocrine dysregulation, inflammatory cytokines, metabolic health, systemic inflammation

## Abstract

Chronic pain is increasingly recognised as a systemic disorder with effects that extend beyond nociception to influence immune, endocrine, cardiovascular, and metabolic systems. Sustained nociceptive activity is associated with low‑grade inflammation and neuroendocrine dysregulation, characterised by elevated pro‑inflammatory cytokines and altered hormonal signalling. These changes are consistently associated with endothelial dysfunction, hypertension, insulin resistance, dyslipidaemia, and increased cardiometabolic risk. This narrative review synthesises evidence from human and preclinical studies published between 2000 and 2025 to examine the immune‑endocrine mechanisms linking chronic pain with cardiovascular and metabolic dysfunction. A structured narrative approach was used, prioritising human studies for clinical relevance and animal studies for mechanistic insight. Evidence suggests that inflammatory cytokines (e.g., interleukin-1β (IL‑1β), interleukin‑6 (IL‑6), tumour necrosis factor-alpha (TNF‑α)) and endocrine pathways, including the hypothalamic-pituitary-adrenal (HPA) axis, insulin signalling, adipokines, and the renin-angiotensin-aldosterone system, interact bidirectionally to perpetuate pain and cardiometabolic disease. However, many findings are associative, and convergent experimental data support plausible causal pathways. Recognising chronic pain as part of a multisystem pathophysiological continuum has important implications for early cardiometabolic screening and integrated clinical management.

## Introduction and background

Chronic pain affects approximately 15-30% of adults globally, with variation by age, sex, socioeconomic status, and geographical region, and is increasingly recognised as a complex, multidimensional condition rather than a purely sensory experience [1-3]. Contemporary frameworks conceptualise chronic pain as a biopsychosocial and physiological disorder arising from the interaction of neural, immune, endocrine, and behavioural processes. Beyond its substantial impact on quality of life, chronic pain is associated with increased cardiovascular morbidity and mortality, including higher prevalence of hypertension, coronary artery disease, and heart failure. In parallel, chronic pain frequently co-exists with metabolic disorders such as obesity, insulin resistance, and type 2 diabetes mellitus, contributing to a multidimensional health burden that spans multiple physiological systems [1-3]. Epidemiological studies suggest that chronic pain is associated with a 20-50% increased risk of adverse cardiovascular outcomes in population-based cohorts, even after partial adjustment for traditional risk factors [1-3].

Emerging evidence indicates that inflammatory cytokines and endocrine regulatory pathways play central roles in mediating the relationship between chronic pain and cardiometabolic dysfunction. Persistent activation of nociceptive pathways stimulates immune responses and promotes the release of pro-inflammatory mediators, including interleukin-1β (IL‑1β), interleukin‑6 (IL‑6), and tumour necrosis factor-alpha (TNF‑α) [4]. Human studies associate elevations in these mediators with endothelial dysfunction, impaired vascular homeostasis, and oxidative stress, while experimental models provide mechanistic evidence that cytokine signalling can directly disrupt endothelial integrity and promote atherogenic processes. Concurrent activation of the hypothalamic-pituitary-adrenal (HPA) axis and the sympathetic nervous system alters cortisol and catecholamine secretion, contributing to an endocrine imbalance that may further exacerbate inflammatory and metabolic dysregulation [5].

Together, pain, inflammation, and endocrine dysregulation form a closely interconnected pathophysiological triad with important cardiovascular and metabolic consequences. Chronic low-grade inflammation has been associated with insulin resistance and dyslipidaemia, while prolonged cortisol exposure is linked to impaired glucose metabolism and increased visceral adiposity, an established risk factor for cardiometabolic disease. However, despite growing mechanistic plausibility, the temporal and causal relationships linking pain-induced immune and endocrine disruptions to downstream cardiovascular and metabolic outcomes remain incompletely characterised, with much of the existing human evidence being associative in nature [6].

Accordingly, this narrative review synthesises current human and preclinical evidence on the role of inflammatory cytokines and endocrine dysregulation in pain-related cardiovascular dysfunction, with particular attention to metabolic health implications. The review aims to clarify proposed mechanistic pathways, highlight key gaps in the existing literature, and outline priorities for future longitudinal research and integrated clinical management within the pain-inflammation-endocrine-cardiometabolic axis.

## Review

Methods

Structured Narrative Approach

This review was conducted as a narrative synthesis using a structured narrative approach to enhance transparency and methodological rigour while remaining appropriate for a mechanism-focused review. Rather than estimating pooled effect sizes, the aim was to integrate evidence across immune, endocrine, cardiovascular, and metabolic domains to identify convergent biological pathways linking chronic pain to cardiometabolic dysfunction. Consistent with recommended guidance for narrative reviews, study selection and synthesis were guided by conceptual relevance, mechanistic plausibility, and consistency of findings across human and preclinical models [7,8].

Search Strategy

A comprehensive literature search was performed to identify relevant studies examining associations between inflammatory cytokines, endocrine dysregulation, pain, cardiovascular function, and metabolic health. Peer-reviewed articles were searched in PubMed, Scopus, Web of Science, and Google Scholar, covering publications from January 2000 to September 2025. To minimise omission of key evidence, reference lists of highly cited and conceptually important articles were manually screened to identify additional eligible studies. Search strategies were adapted to the indexing structure and functionality of each database to optimise the retrieval of relevant studies; however, full database-specific search strings were not retained in a reproducible format and are therefore not provided as supplementary material.

Search Terms

Medical Subject Headings (MeSH) and free-text keywords were used individually and in combination, including pain, chronic pain, inflammation, inflammatory cytokines, interleukin-6 (IL-6), tumour necrosis factor-alpha (TNF-α), endocrine dysregulation, hypothalamic-pituitary-adrenal (HPA) axis, cortisol, cardiovascular dysfunction, metabolic syndrome, insulin resistance, and metabolic health. Boolean operators AND and OR were applied to refine and combine search results.

Inclusion and Exclusion Criteria

Studies were included if they examined relationships between pain and inflammatory or endocrine markers and reported cardiovascular or metabolic outcomes or provided mechanistic insight into these interactions. Eligible studies were required to be published in English, appear in peer-reviewed journals, and employ clearly described human or animal experimental or observational methodologies. Studies were excluded if they did not directly address the pain cytokine-endocrine-cardiometabolic axis. Abstracts, conference proceedings, non-peer-reviewed articles, commentaries, and studies lacking sufficient methodological or mechanistic detail were excluded.

Data Extraction and Synthesis

Data were extracted on study design, population characteristics, pain phenotype, inflammatory and endocrine biomarkers assessed, and reported cardiovascular or metabolic outcomes. Findings were synthesised thematically using narrative integration to identify recurring patterns and convergent mechanisms linking pain-induced inflammation and endocrine dysregulation to cardiometabolic impairment. Human studies were prioritised for clinical relevance, while animal and preclinical studies were used primarily to support causal and mechanistic inference. Where evidence was heterogeneous, conclusions were framed conservatively, with explicit distinction between associative and experimental findings. Study identification, screening, and data extraction were conducted by a single reviewer; therefore, no formal process for resolving disagreements between reviewers was required. Temporal relationships and dose-response patterns between inflammatory or endocrine markers and cardiometabolic outcomes were not systematically assessed in this review; however, where longitudinal or experimental evidence suggested potential temporal sequencing, this was noted narratively, and the overall lack of temporal resolution in the literature is acknowledged as a key limitation.

Methodological limitations

As a narrative review, no formal risk-of-bias assessment or quantitative meta-analysis was conducted. Consequently, this approach inherently carries a risk of selection and interpretive bias, and the synthesis is susceptible to heterogeneity in study design, populations, and biomarker measurement methods. In addition, residual confounding from factors such as medication use, physical inactivity, sleep disturbance, and psychological comorbidity may influence reported associations. These limitations were considered when interpreting findings and in highlighting priorities for further longitudinal and mechanistic research.

Pain, inflammatory cytokines, and cardiovascular dysfunction

Pain, whether acute or chronic, elicits complex physiological responses that extend beyond nociceptive processing to involve the immune, endocrine, and cardiovascular systems. Acute pain triggers short-term inflammatory and immune responses that facilitate tissue repair and recovery. In contrast, chronic pain, arising from neuropathic injury, musculoskeletal inflammation, or visceral pathology, is commonly associated with persistent low-grade systemic inflammation. Prolonged activation of nociceptive pathways promotes sustained immune cell activation and continuous release of pro-inflammatory cytokines, including IL-1β, IL-6, TNF-α, and IL-17 [9-12].

These cytokines disrupt vascular homeostasis through multiple mechanisms: they induce endothelial activation, increase vascular permeability, reduce nitric oxide bioavailability, and promote vascular stiffness and microcirculatory dysfunction. TNF-α additionally upregulates endothelial adhesion molecules such as vascular cell adhesion molecule-1 (VCAM-1) and intercellular adhesion molecule-1 (ICAM-1), facilitating leukocyte adhesion and transmigration into the vascular wall. Over time, these inflammatory changes contribute to atherosclerotic progression, myocardial remodelling, and the deterioration of cardiac function [12-14].

Endothelial dysfunction in chronic pain appears to arise through both direct cytokine-mediated mechanisms, including reduced nitric oxide bioavailability and increased expression of endothelial adhesion molecules, and indirect pathways involving autonomic imbalance, sympathetic overactivity, and pain-related metabolic disturbances such as insulin resistance and adipokine dysregulation [11,12].

Human observational studies demonstrate that elevated IL-6 and C-reactive protein (CRP) levels are associated with increased risk of hypertension, subclinical atherosclerosis, coronary artery disease, carotid intima-media thickness, and heart failure in individuals with chronic pain. While these findings are primarily associative, experimental animal models provide complementary mechanistic evidence, showing that elevation of pro-inflammatory cytokines induces endothelial dysfunction, oxidative stress, and myocardial remodelling, whereas cytokine inhibition attenuates these effects [13,14].

Pain phenotypes differ in immune-endocrine profiles and cardiometabolic implications. Inflammatory pain conditions often show stronger peripheral cytokine elevations and clearer associations with metabolic syndrome. Neuropathic pain may involve neuroimmune mechanisms and is frequently intertwined with metabolic disorders such as diabetes. Centralised pain syndromes, including fibromyalgia, tend to exhibit altered stress-axis regulation and autonomic imbalance, with cardiometabolic risk potentially mediated more by physical inactivity, sleep disturbance, and mood disorders than by high peripheral cytokine levels alone. Considerable overlap exists between phenotypes, and heterogeneity should be acknowledged in both research and clinical assessment [10,11].

Collectively, these convergent lines of evidence identify inflammation as a central biological pathway linking chronic pain to cardiovascular dysfunction. They underscore the need for longitudinal human studies to clarify causal relationships and to determine whether targeted modulation of cytokine signalling may reduce cardiometabolic risk in chronic pain populations.

Available evidence suggests that early cardiovascular changes in chronic pain are characterised by endothelial dysfunction, autonomic imbalance, and low-grade metabolic disturbance, which may precede the development of later overt cardiovascular disease such as hypertension, atherosclerosis, and heart failure; however, the precise temporal sequencing of these processes remains incompletely characterised due to a lack of longitudinal human studies [14].

Endocrine dysregulation beyond the HPA axis

HPA Axis and Sympathetic Activation

Chronic pain is associated with sustained activation of the HPA axis and the sympathetic nervous system, resulting in prolonged exposure to cortisol and catecholamines. While acute activation of these stress pathways is adaptive, persistent hypercortisolaemia and sympathetic overactivity are associated with hypertension, arterial stiffness, impaired glucose metabolism, and increased visceral adiposity, thereby contributing to elevated cardiometabolic risk [11]. Chronic sympathetic activation may further exacerbate cardiovascular strain through increased vascular tone, autonomic imbalance, and oxidative stress [10].

Heterogeneity by Sex, Age, and Pain Duration

Endocrine and inflammatory responses to chronic pain are heterogeneous and may vary according to sex, age, and duration of pain exposure, with evidence suggesting sex-specific differences in stress hormone regulation, age-related alterations in neuroendocrine responsiveness, and adaptive or maladaptive endocrine changes with prolonged pain duration. Such heterogeneity may partially explain variability in cardiometabolic risk among chronic pain populations and warrants further investigation [4,5,11].

Relative Contribution of HPA Axis Dysregulation Versus Sympathetic Activation

Both HPA axis dysregulation and sustained sympathetic activation appear to contribute to cardiometabolic complications in chronic pain; however, available evidence suggests that sympathetic overactivity plays a more direct role in hypertension, vascular tone, and myocardial strain, whereas HPA axis alterations may exert broader modulatory effects through metabolic, inflammatory, and neuroendocrine pathways. The relative dominance of these mechanisms likely varies across individuals and pain phenotypes and remains incompletely delineated in human studies [10,11].

Sustained Baseline Shifts Versus Stress-Reactive Endocrine Changes

Endocrine alterations observed in chronic pain may reflect a combination of sustained baseline dysregulation and repeated stress-reactive responses to ongoing or recurrent pain episodes. While acute pain triggers transient activation of the HPA axis and sympatho-adrenal system, persistent pain appears to promote longer-term alterations in cortisol and catecholamine regulation, although the relative contribution of baseline shifts versus repeated reactivity remains incompletely characterised in human studies [11].

Circadian Rhythm Disruption and Cardiovascular Risk

Chronic pain is also associated with disruption of normal circadian rhythms governing cortisol and catecholamine secretion, including blunted diurnal variation and impaired nocturnal recovery. Such circadian dysregulation may contribute to sustained sympathetic tone, metabolic impairment, and increased cardiovascular risk, representing an additional pathway linking chronic pain to cardiometabolic dysfunction [10,11].

Insulin signalling and glucose homeostasis

Pro-inflammatory cytokines, particularly TNF-α and IL-6, interfere with insulin receptor signalling pathways, promoting insulin resistance and compensatory hyperinsulinaemia. In parallel, sustained cortisol exposure enhances hepatic gluconeogenesis and reduces peripheral glucose uptake, contributing to glucose intolerance and metabolic dysregulation. These immune-endocrine interactions provide a mechanistic link between pain-related stress responses and the development of insulin resistance and type 2 diabetes mellitus [14].

Adipokines and renin-angiotensin-aldosterone system

Chronic pain-related inflammation is also associated with adipokine imbalance, characterised by elevated leptin levels and reduced adiponectin concentrations. This dysregulation promotes vascular inflammation, endothelial dysfunction, and impaired insulin sensitivity [15,16]. Additionally, chronic stress and sympathetic activation may stimulate the renin-angiotensin-aldosterone system, leading to increased vascular tone, sodium retention, oxidative stress, and further cardiometabolic burden [16].

Intersection and bidirectionality of pain and cardiometabolic disease

Chronic pain induces a dynamic interplay between inflammatory and endocrine systems, forming a self-sustaining loop that amplifies cardiovascular risk. In this maladaptive feedback cycle, pro-inflammatory cytokines such as IL-6 activate neuroendocrine stress pathways, including the HPA axis, while hormonal dysregulation in turn modulates immune activity and cytokine expression. Mechanistically, TNF-α and IL-6 impair insulin receptor signalling, contributing to reduced insulin uptake and hyperinsulinaemia, both of which promote endothelial dysfunction. Elevated catecholamine levels further aggravate vascular injury by stimulating vascular smooth muscle proliferation and fibrosis. Together, chronic inflammation and sympathetic overactivity impair autonomic regulation, including reduced baroreceptor sensitivity and heart rate variability, thereby increasing susceptibility to hypertension, arrhythmias, and broader cardiovascular instability.

Clinical observational studies, including population-based cohorts and clinical samples of adults with chronic musculoskeletal and inflammatory pain, demonstrate that patients with chronic pain exhibit elevated circulating IL-6 and cortisol levels that correlate with subclinical markers of atherosclerosis, such as increased carotid intima-media thickness [14]. In contrast, preclinical animal models provide mechanistic support, showing that experimental disruption of IL-6 signalling alters insulin sensitivity and endothelial function, underscoring the integrative role of inflammatory-endocrine cross-talk in cardiovascular pathology [15]. Importantly, this relationship is bidirectional: cardiometabolic disease states such as obesity, insulin resistance, and endothelial dysfunction can further amplify systemic inflammation and pain sensitisation, reinforcing chronic pain states rather than pain acting solely as an upstream driver [17,18,19].

Confounding factors and effect modifiers

Available epidemiological studies suggest that the association between chronic pain and cardiovascular outcomes is attenuated but not fully eliminated after adjustment for shared risk factors such as age, socioeconomic deprivation, physical inactivity, and comorbid depression or anxiety, indicating both independent and mediated effects. Interpretation of immune endocrine markers in chronic pain is influenced by several confounders, including analgesic and steroid use, physical inactivity, sleep disturbance, depression, and anxiety. These factors can independently alter cytokine levels, hormonal profiles, and cardiometabolic risk and should be carefully accounted for in both research and clinical practice.

Table 1 summarises the convergent mechanisms through which inflammatory and endocrine mediators contribute to pain-related cardiovascular dysfunction. It provides a concise overview of key cytokine endocrine interactions and their systemic effects to help readers visualise the multisystem interplay without repeating the main text.

**Table 1:** Key inflammatory and endocrine mediators linking chronic pain to cardiovascular and metabolic dysfunction Pathways listed represent key examples rather than an exhaustive summary. Evidence type reflects the predominant source supporting each pathway (preclinical, human observational, or interventional). Overall evidence strength was judged narratively based on consistency of findings, biological plausibility, and availability of human data; most pathways are supported primarily by observational and preclinical evidence, with limited interventional confirmation. IL-1β: interleukin-1 beta; IL-6: interleukin-6; TNF-α: tumour necrosis factor-alpha; IL-17: interleukin-17; CRP: C-reactive protein; ACTH: adrenocorticotropic hormone; HPA axis: hypothalamic-pituitary-adrenal axis; FFA: free fatty acid

Pathway	Principal mediators	Mechanistic effects	Cardiovascular outcomes	Metabolic outcomes
Inflammatory	IL-1β, IL-6, TNF-α, IL-17, CRP	Endothelial activation, oxidative stress, leukocyte adhesion	Endothelial dysfunction, hypertension, atherosclerosis	Insulin resistance, dyslipidaemia [20]
Endocrine (HPA axis)	Cortisol, ACTH	Chronic hypercortisolemia, receptor desensitisation, altered circadian rhythm	Arterial stiffness, hypertension	Visceral adiposity, glucose intolerance [14]
Sympathetic activation	Epinephrine, norepinephrine	Vasoconstriction, oxidative stress, cardiac workload	Tachycardia, myocardial strain	Elevated glucose and FFA levels [10]
Adipokine imbalance	Leptin, adiponectin	Impaired insulin signalling, lipid accumulation	Vascular inflammation	Insulin resistance, obesity [18]
Cross-talk	IL-6-cortisol loop	Mutual amplification of inflammation and hormonal dysregulation	Cardiometabolic dysfunction	Metabolic syndrome [19]

Implications for metabolic health

Metabolic alterations represent a critical interface through which inflammatory and endocrine dysregulation associated with chronic pain may influence cardiovascular risk. Sustained activation of inflammatory pathways is consistently associated with insulin resistance, dyslipidaemia, and visceral adiposity, key components of the metabolic syndrome. Pro-inflammatory cytokines, particularly TNF-α, can suppress adiponectin secretion, thereby reducing insulin sensitivity and impairing lipid metabolism [21]. In parallel, chronic activation of the HPA axis and prolonged cortisol exposure are associated with increased hepatic gluconeogenesis, central fat accumulation, and glucose intolerance, further contributing to metabolic dysregulation.

Endothelial dysfunction and autonomic imbalance may compound these effects. Impaired endothelial function can reduce peripheral glucose uptake, while sustained sympathetic nervous system activation is associated with elevated fasting glucose levels and increased circulating free fatty acids. Collectively, these immune, endocrine, and autonomic alterations may contribute to a bidirectional feedback loop in which metabolic dysfunction amplifies pain and inflammatory signalling, potentially leading to progressive impairment of physiological homeostasis. Epidemiological studies support these associations, demonstrating that individuals with chronic pain are more likely to exhibit insulin resistance and features of metabolic syndrome [22,23]. In addition, elevated circulating levels of CRP and cortisol have been associated with higher body mass index and poorer glycaemic control in chronic pain populations.

Taken together, available evidence suggests that chronic pain, cardiovascular dysfunction, and metabolic dysregulation likely interact as components of a shared pathophysiological continuum rather than functioning as entirely isolated conditions. This integrated perspective helps explain the increased risk of type 2 diabetes mellitus and atherosclerotic cardiovascular disease observed among individuals living with chronic pain [22].

Clinical and translational perspectives

Recognising chronic pain as a multisystem condition characterised by immune, endocrine, cardiovascular, and metabolic dysregulation has important implications for clinical practice. Evidence synthesised in this structured narrative review highlights convergent biological pathways through which persistent pain-related inflammation and endocrine imbalance are associated with increased cardiometabolic risk. By integrating findings from human observational studies and preclinical mechanistic models, this review underscores the potential value of early cardiometabolic surveillance in individuals living with chronic pain, particularly those with long-standing or multisite pain syndromes [9,11,24].

From a translational perspective, the feasibility and clinical utility of proposed immune and endocrine biomarkers vary considerably. Widely available and cost-effective measures, such as blood pressure, body mass index, waist circumference, lipid profile, HbA1c, and CRP, are currently the most practical tools for routine cardiometabolic screening in chronic pain populations. In contrast, cytokines such as IL-6 and TNF-α, as well as detailed cortisol or catecholamine profiling, are subject to greater biological variability, higher assay cost, and limited accessibility across clinical settings. Consequently, while these biomarkers provide valuable mechanistic insight, many should presently be regarded as exploratory or associative rather than independently prognostic in routine clinical care.

Recognition of the interconnected disruption of endocrine function, cardiovascular regulation, and pain-induced inflammation also highlights the importance of distinguishing evidence-supported interventions from those that remain theoretical or extrapolated. Lifestyle modification, optimisation of cardiometabolic risk factors, and multidisciplinary pain management strategies are supported by evidence across both chronic pain and cardiometabolic conditions. In contrast, targeted anti-cytokine or hormonal interventions are largely extrapolated from related inflammatory or endocrine disorders and have not been systematically evaluated in chronic pain populations. Such approaches may carry potential risks, including immunosuppression, infection susceptibility, metabolic disturbance, or unintended neuroendocrine effects, and therefore cannot yet be routinely recommended.

Accordingly, an integrated clinical approach is likely to require prioritisation and sequencing of multimodal interventions, with initial emphasis on modifiable risk factors such as physical inactivity, obesity, sleep disturbance, and psychological comorbidity, followed by pharmacological or specialist interventions where clinically indicated. Examples of non-pharmacological strategies that may modulate immune, endocrine, and autonomic pathways include cognitive behavioural therapy (CBT), mindfulness-based stress reduction (MBSR), and emerging approaches targeting autonomic regulation, such as vagal nerve modulation, which have been shown to influence stress responsivity, inflammatory signalling, and cardiometabolic risk factors in chronic pain and related conditions [11,24,25].

**Figure 1:**
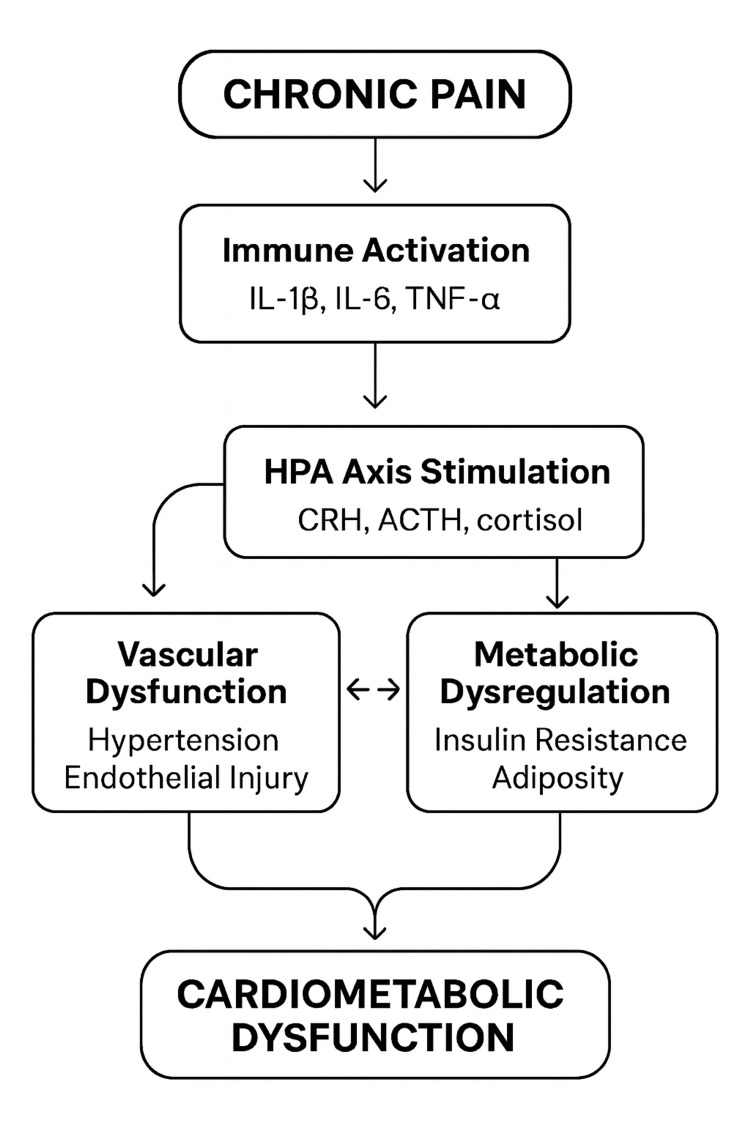
Integrated model of pain-induced immune-endocrine interactions leading to cardiometabolic dysfunction Integrated schematic illustrating immune-endocrine interactions underlying chronic pain and cardiometabolic dysfunction. Solid arrows indicate pathways supported by relatively consistent human observational and/or experimental evidence, whereas dashed arrows denote hypothesised or emerging links for which evidence is currently limited or predominantly indirect. Pathways primarily supported by preclinical (animal or in vitro) studies are annotated accordingly, while links informed mainly by human observational data are indicated where applicable. The figure emphasises bidirectional interactions between inflammatory cytokines, neuroendocrine stress pathways, autonomic regulation, and metabolic dysfunction that may collectively contribute to increased cardiovascular risk in chronic pain populations. IL-1β: interleukin-1 beta; IL-6: interleukin-6; TNF-α: tumour necrosis factor-alpha; CRH: corticotropin-releasing hormone; ACTH: adrenocorticotropic hormone; HPA: hypothalamic-pituitary-adrenal axis The figure was created by the authors.

Figure 1 presents an integrated schematic of immune-endocrine interactions underlying chronic pain and its cardiometabolic consequences. It illustrates the bidirectional pathways through which persistent nociceptive activity contributes to cardiovascular and metabolic dysfunction. Chronic nociceptive stimulation activates immune cells, promoting the release of pro-inflammatory cytokines such as IL-1, IL-6, and TNF-α, which disrupt vascular homeostasis and induce endothelial injury. Concurrent activation of the HPA axis and the sympathetic-adrenal-medullary system elevates cortisol, epinephrine, and norepinephrine levels, fostering hypertension, oxidative stress, and dysregulated glucose and lipid metabolism. Reciprocal reinforcement between hormonal and cytokine pathways sustains low-grade inflammation and endocrine imbalance, culminating in cardiometabolic disturbances including endothelial dysfunction, insulin resistance, visceral adiposity, and elevated cardiovascular risk [10,19,24].

Despite these advances, important gaps remain in understanding how immune and endocrine mechanisms interact dynamically over time in chronic pain. Future research should prioritise longitudinal studies integrating molecular profiling with clinical outcomes, alongside interventional trials designed to test whether modifying immune-endocrine pathways can meaningfully reduce cardiometabolic risk. Inclusion of diverse populations and sex-specific analyses will also be critical for clarifying individual susceptibility and differential responses to intervention [25].

Remaining knowledge gaps and future directions

Despite increasing recognition of chronic pain as a condition involving immune, endocrine, cardiovascular, and metabolic dysregulation, several important gaps remain in the current evidence base. Chronic pain prevalence is commonly reported to affect approximately one in five adults worldwide; however, population-specific prevalence ranges and representative quantitative estimates of associated cardiovascular risk remain incompletely characterised, limiting accurate epidemiological anchoring of the problem across diverse populations [1-3]. Moreover, although associations between chronic pain and adverse cardiovascular outcomes are frequently described, it remains unclear to what extent these relationships are independent of shared risk factors, including age, socioeconomic status, physical inactivity, and comorbid depression or anxiety, as many observational studies do not fully disentangle confounding from mediation [4,5].

Beyond the extensively studied cytokines IL-1β, IL-6, and TNF-α, emerging inflammatory mediators such as IL-17 and CRP are increasingly implicated in chronic pain and cardiometabolic disease; however, their precise roles remain insufficiently defined [11,22]. In addition, innate immune mechanisms, including inflammasome-related pathways, have received comparatively little attention in pain-related cardiometabolic research, despite growing evidence linking these pathways to vascular and metabolic dysfunction. While endocrine systems involving insulin signalling, adipokines, and the renin-angiotensin-aldosterone system are discussed within the existing literature, their temporal sequencing and relative contribution across different chronic pain phenotypes remain poorly delineated [15-18].

Importantly, when mechanisms are described as "remaining incompletely characterised", this reflects several specific limitations in the literature, including a lack of longitudinal studies capable of establishing temporal and causal relationships, a scarcity of mechanistic human studies integrating repeated biomarker assessments with clinical outcomes, and limited interventional evidence evaluating whether modulation of immune or endocrine pathways can meaningfully reduce cardiometabolic risk in chronic pain populations [7,8,14]. Finally, although this review highlights the potential value of early cardiometabolic screening, how immune-endocrine profiling should be incorporated into prevention strategies, risk stratification models, or therapeutic decision-making frameworks has yet to be systematically evaluated [23-25].

## Conclusions

Chronic pain is not merely a neurological disorder but a systemic condition with far-reaching effects on cardiovascular and metabolic health. Persistent nociceptive input drives the sustained release of pro-inflammatory cytokines, including IL-1, IL-6, and TNF-α, which contributes to endothelial dysfunction and vascular impairment. Concurrent dysregulation of the HPA axis and chronic sympathetic activation leads to abnormal secretion of cortisol and catecholamines, exacerbating vascular stress and disrupting glucose and lipid metabolism. Together, these mechanisms form a pathophysiological interface linking chronic pain to cardiometabolic disease, increasing susceptibility to conditions such as insulin resistance, obesity, type 2 diabetes mellitus, hypertension, and coronary artery disease.

These observations have important clinical implications. Chronic pain should be approached as an integrated, multisystem disorder in which therapeutic strategies address not only nociceptive symptoms but also underlying inflammatory and endocrine dysregulation. Routine assessment of cardiometabolic risk factors and relevant biomarkers can enhance the early detection of systemic complications and inform personalised treatment plans.

Although much of the human evidence remains associative, convergent experimental and preclinical data support plausible mechanistic pathways connecting pain, immune activation, hormonal imbalance, and cardiovascular dysfunction. Longitudinal and mechanistic clinical studies are needed to clarify causal relationships and identify optimal intervention points. Such research will be critical for developing targeted, multisystem interventions capable of interrupting the self-perpetuating cycle of chronic pain, inflammation, endocrine dysregulation, and cardiometabolic risk.

In summary, recognising chronic pain as a systemic, multisystem condition underscores the need for integrated, multidisciplinary management strategies. Addressing the inflammatory and endocrine mediators of pain-induced cardiovascular and metabolic dysfunction holds promise for improving quality of life, reducing long-term cardiometabolic morbidity, and optimising overall health outcomes.
